# Endothelial Nitric Oxide Synthase (*eNOS*) *4b/a* Gene Polymorphisms and Coronary Artery Disease: Evidence from a Meta-Analysis

**DOI:** 10.3390/ijms15057987

**Published:** 2014-05-07

**Authors:** Yujiao Yang, Kang Du, Zhengxia Liu, Xiang Lu

**Affiliations:** Department of Geriatrics, the Second Affiliated Hospital, Nanjing Medical University, Nanjing 210029, Jiangsu, China; E-Mails: yangyujiao626@126.com (Y.Y.); dukang@njmu.edu.cn (K.D.); liuzx0503@gmail.com (Z.L.)

**Keywords:** endothelial nitric oxide synthase (eNOS), coronary artery disease (CAD), polymorphism, meta-analysis

## Abstract

A variety of studies have suggested that the 4b/a polymorphism in the endothelial nitric oxide synthase (*eNOS*) was associated with coronary artery disease (CAD) risk. However, the data remain conflicting. The aim of the present meta-analysis was to estimate the overall association between risk of CAD and *eNOS 4b/a* polymorphism. Case-control, cohort or cross-sectional studies evaluating the association between *eNOS 4b/a* polymorphism and CAD susceptibility were systematically identified in PubMed up to 31 October 2013. Pooled odds ratios (OR) and corresponding 95% confidence intervals (CIs) were calculated to assess the association in overall and subgroup analyses. A total of 10,617 cases and 8302 controls from 37 studies were included in the study. The results of overall analysis revealed significant positive associations between CAD risk and *eNOS 4b/a* polymorphism in homozygote comparisons (OR = 1.47, 95% CI = 1.16–1.87), heterozygote comparisons (OR = 1.14, 95% CI = 1.02–1.27) and dominant models (OR = 1.18, 95% CI = 1.06–1.33). In subgroup analyses, similar associations were identified in African individuals, as determined using population-based source subgroups and noted in small-and-moderate sample size subgroups (case sample size or control sample size <500). The current meta-analysis revealed that *eNOS 4b/a* polymorphisms could be a risk factor for developing CAD, particularly in African populations and population-based subgroups.

## 1. Introduction

Coronary artery disease (CAD) also known as coronary heart disease (CHD), is the leading cause of death and disability worldwide [1,2]. CAD is associated with genetic and environmental factors, as well as their interactions [3]. Confirmed risk factors for CAD include hypercholesterolemia, hypertension, smoking, and diabetes [4]. However, in addition to modifiable risk factors, genetic factors can also predispose individuals to CAD. It has been estimated that genetic risk factors explain approximately 20%–60% of CAD cases [5].

Nitric oxide (NO) is an important atheroprotective mediator that helps mediate endothelium-dependent vasodilatation. Abnormalities in NO generation could play an important role in the pathophysiology of CAD [6]. NO is synthesized from l-arginine by the action of nitric oxide synthase (NOS). There are at least three isoenzymes of NOS: inducible NOS, neuronal NOS, and endothelial NOS (eNOS) [7]. The *eNOS* gene is located on chromosome 7q35–q36 and is sized 21 kilobases (kb) [8]. A number of studies have suggested that polymorphisms in the *eNOS* gene affected NO availability and were associated with CAD morbidity. One of the most clinically relevant *eNOS* variants forms 27-basepair (bp) tandem repeats in *intron 4 (4b/a).* The *eNOS 4b* allele contains five repeats of the 27 bp sequence, whereas *eNOS 4a*, which is the rare allele, carries a deletion of one of the first three of these repeats [9].

The association between *eNOS 4b/a* gene polymorphisms and CAD remains controversial, and the results of two previous meta-analyses were inconsistent [10,11]. In 2004, Casas *et al.* performed a meta-analysis of 16 studies and found an increased risk of ischemic heart disease (IHD) in individuals carrying the homozygous a allele compared with the b allele [10]. However, a subsequent meta-analysis in 2010 reported conflicting data, where *eNOS 4b/a* did not significantly increase the risk of developing CAD [11]. A large number of new case-control studies assessing the association between *eNOS 4b/a* polymorphisms and the CAD risk have been published since 2010. Therefore, we performed a meta-analysis using a more complete database to clarify the association between *eNOS 4b/a* polymorphisms and CAD risk.

## 2. Results and Discussion

### 2.1. Characteristics of Included Studies

**Figure 1.**
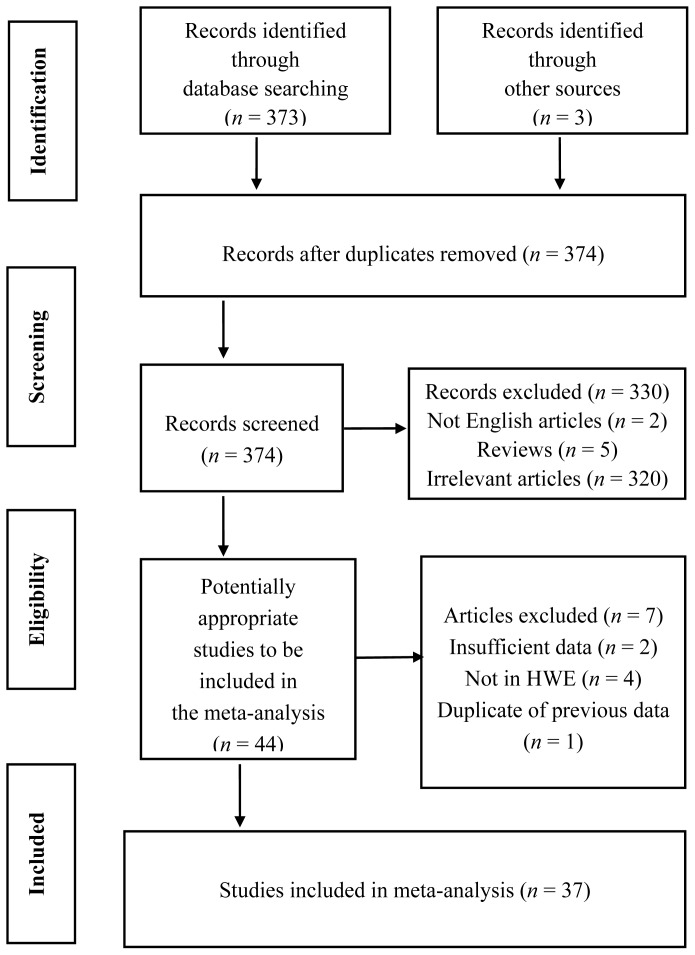
Flow diagram of study identification.

A total of 373 papers were initially identified during the literature search and from other sources; 37 of these met the inclusion criteria for this analysis [9,12–47]. A flow chart describing the literature search and study identification is shown in Figure 1. The meta-analysis of the *4b/a* polymorphism included 10,617 CAD cases and 8302 controls. The study characteristics are summarized in Table 1. The number of subjects in the studies varied considerably (range from 40 to 1265 in the case group and from 34 to 620 in the control group). Among the 37 case-control studies, 11 studies included Asian population [15–25], and 26 assessed non-Asians [9,12–14,26–47]. Controls were mainly matched in terms of gender and age. Fourteen studies were hospital-based [16–18,20,21,25,28,37–40,42,43,45], and 20 were population-based [9,12–15,19,22,23,26,27,29–34,36,41,46,47]. Thirty studies were performed using <500 subjects in the case group [12–27,29,33–36,38–47] and 33 were conducted with <500 subjects in the control group [9,12–15,17–29,32,34–47]. Furthermore, there were 20 studies of CAD [9,18,20,22,23,25,29,30,32,34–40,42–44,46], four of CHD [17,26–28], three of acute myocardial infarction [15,19,45], six of myocardial infarction (MI) [12–14,16,31,41], and four of acute coronary syndrome [21,24,33,47].

### 2.2. Meta-Analysis Results

The relationship between *eNOS 4b/a* polymorphisms and CAD risk are summarized in Table 2. Overall, significant positive associations between CAD risk and *eNOS 4b/a* polymorphism were identified in the homozygote comparison (*aa vs. bb*, OR = 1.47, 95% CI = 1.16–1.87, *p*_heterogeneity_ = 0.085), the heterozygote comparison (*ab vs. bb*, OR = 1.14, 95% CI = 1.02–1.27, *p*_heterogeneity_ < 0.001) and the dominant model (*ab* + *aa vs. bb*, OR = 1.18, 95% CI = 1.06–1.33, *p*_heterogeneity_ < 0.001).

**Figure 2.**
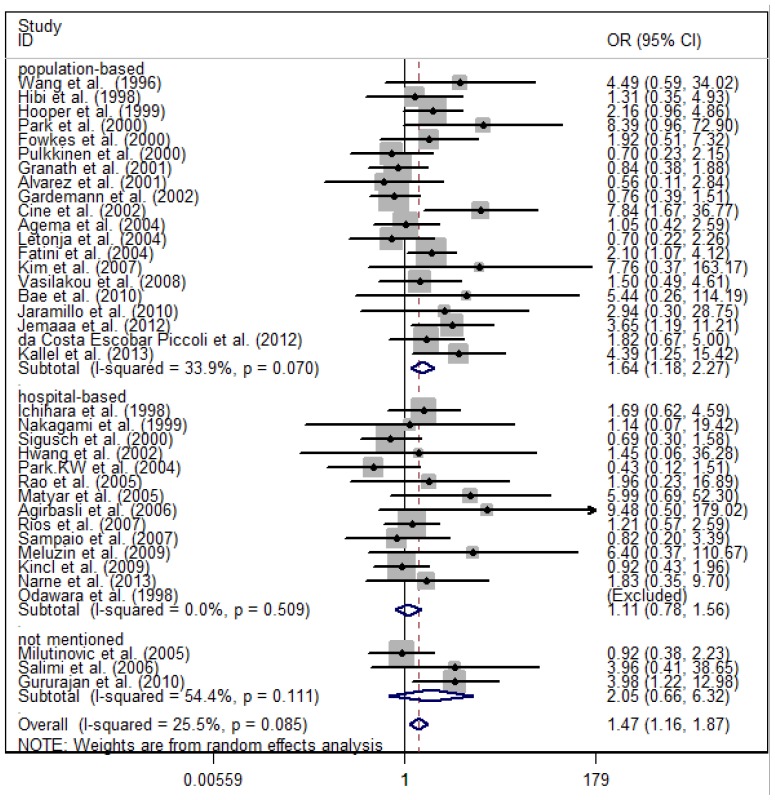
Forest plot of CAD risk associated with the *eNOS 4b/a* polymorphism by source of control (homozygote comparison).

In a stratified analysis by the source of the control population, significantly increased CAD risk was detected in the population-based subgroup of all the three genetic models (OR = 1.64, 95% CI = 1.18–2.27, *p* = 0.070 for the homozygote comparison; OR = 1.16, 95% CI = 1.00–1.35, *p* < 0.001 for the heterozygote comparison; and OR = 1.21, 95% CI = 1.05–1.40, *p* < 0.001 for the dominant model). There was no significantly increased CAD risk in the hospital-based subgroup (OR = 1.11, 95% CI =0.78–1.56, *p* = 0.509 for the homozygote comparison; OR = 1.04, 95% CI = 0.88–1.23, *p* = 0.072 for the heterozygote comparison; and OR = 1.07, 95% CI = 0.89–1.27, *p* = 0.027 in the dominant model) (Figure 2).

The data were also stratified into Asian and non-Asian subpopulations. There was a statistically significant association in the non-Asian subgroup in the homozygote comparison (OR = 1.41, 95% CI = 1.08–1.84, *p* = 0.080) and the dominant model (OR = 1.16, 95% CI = 1.02–1.32, *p* < 0.001). Non-Asians were then further stratified into Caucasians, Africans, and others. The populations of an American study and a Brazilian study were both composed of two ethnicities: Caucasians and Africans [39,40]. The different ethnic descents in these studies were considered to be two independent studies during sub-stratified analyses. Statistically significant associations were noted in African populations in three genetic models (homozygote comparison OR = 2.93, 95% CI = 1.71–5.03, *p* = 0.821; heterozygote comparison OR = 1.46, 95% CI = 1.16–1.83, *p* = 0.353; and the dominant model OR = 1.58, 95% CI = 1.27–1.96, *p =* 0.370). Interestingly, significant associations were also found in Asian (OR = 1.82, 95% CI = 1.03–3.20, *p* = 0.316) and “other” (OR = 2.65, 95% CI = 1.37–5.13, *p* = 0.369) populations during the homozygote comparison. In contrast, no statistically significant associations were identified in Caucasian populations (OR = 1.03, 95% CI = 0.80–1.31, *p* = 0.621 in the homozygote comparison; OR = 1.04, 95% CI = 0.90–1.20, *p* = 0.006 in the heterozygote comparison; OR = 1.04, 95% CI = 0.91–1.19, *p* = 0.007 in the dominant model) (Supplementary Figure S1).

The data were then also stratified according to case sample size into large (>500 total cases) and small-and-moderate case sample (<500 total cases) subgroups. Significant associations between *eNOS 4b/a* polymorphisms and CAD risk were observed in the small-and-moderate case sample subgroup (OR = 1.75, 95% CI = 1.35–2.28, *p* = 0.262 in the homozygote comparison; OR = 1.20, 95% CI = 1.07–1.35, *p* = 0.011 in the heterozygote comparison; and OR = 1.26, 95% CI = 1.11–1.42, *p* = 0.002 in the dominant model) but not in the large case sample subgroup. The data were then stratified into large control (>500 control individuals) and small-and-moderate control sample (<500 control individuals) subgroups. Similar to the data from the case sample size analyses, significant associations were observed in the small-and-moderate control sample subgroup (OR = 1.56, 95% CI = 1.19–2.05, *p* = 0.113 for the homozygote comparison; OR = 1.15, 95% CI = 1.01–1.31, *p* < 0.001 for the heterozygote comparison; and OR = 1.20, 95% CI = 1.06–1.36, *p* < 0.001 in the dominant model), but not the large case sample subgroup.

Finally, a subgroup analysis for MI was performed. There were significant associations between *eNOS 4b/a* polymorphisms and CAD risk with the homozygote comparison (OR = 2.30, 95% CI = 1.17–4.51, *p* = 0.020), heterozygote comparison (OR = 1.42, 95% CI = 1.07–1.89, *p* = 0.002), and dominant model (OR = 1.50, 95% CI = 1.09–2.07, *p* < 0.001) in patients with MI.

### 2.3. Evaluation of Heterogeneity

There was minor heterogeneity among studies in the recessive model (*ab* + *bb vs. aa*, *p*_heterogeneity_ = 0.213, *I*^2^ = 15.4%), while the results of heterogeneity tests were significant in the other three genetic models (*aa vs. bb*, *p*_heterogeneity_ = 0.085, *I*^2^ = 25.5%; *ab vs bb*, *p*_heterogeneity_ < 0.001, *I*^2^ = 56.7%; *ab* + *aa vs. bb*, *p*_heterogeneity_ < 0.001, *I*^2^ = 61.1%). The random-effects models were used in all the four genetic models. Meta-regression analyses were then performed to evaluate the extent to which different variables explained the heterogeneity. The results revealed that the heterogeneity could be explained by case sample size (*p* = 0.015) and total sample size (*p* = 0.015) in the homozygote comparison. However, population ethnicity, year of publication, source of control population, total sample size, and outcome were not statistically correlated with heterogeneity (*p* > 0.05).

### 2.4. Sensitivity Analysis and Publication Bias

**Figure 3.**
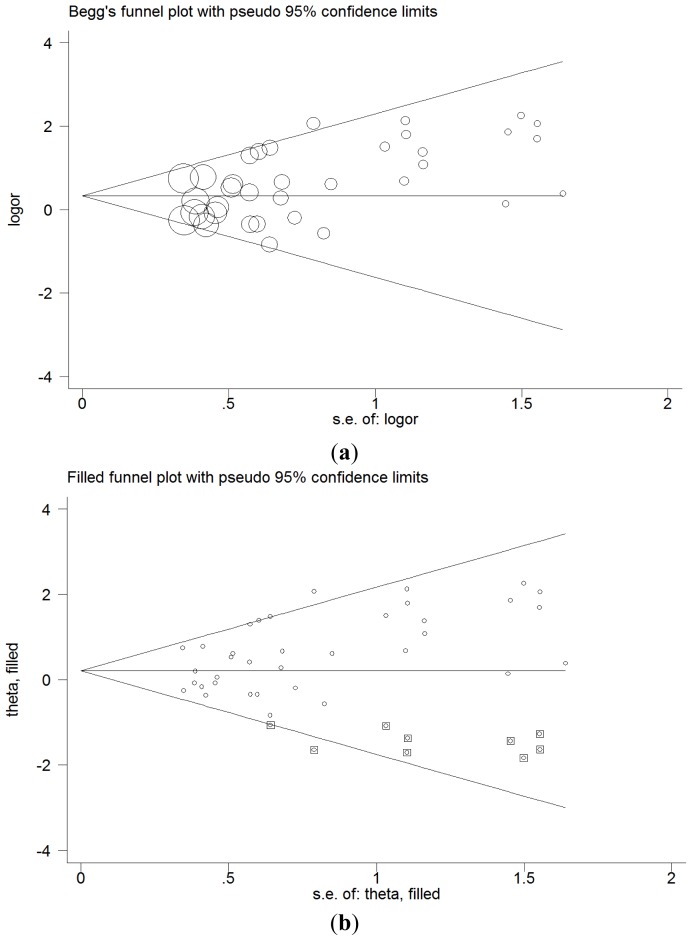
Begg’s funnel plot of publication bias test (homozygote comparison). (**a**) Before trim and fill method; and (**b**) After trim and fill method.

Sensitivity analysis was performed by omitting one study at a time. No significant differences in the data were observed, indicating that the results were statistically reliable (Supplementary Figure S2). The publication bias of the studies was assessed by performing the Begg’s funnel plot and Egger’s test. There was no obvious publication bias in the heterozygote comparison (*ab vs. bb*, Begg’s test *p* = 0.754, Egger’s test *p* = 0.473), and the dominant model (*ab* + *aa vs. bb*, Begg’s test *p* = 0.619, Egger’s test *p* = 0.274). The shape of the funnel plot for the homozygote comparison appeared to be approximately asymmetrical, which was confirmed by the test results (*aa vs. bb*, Begg’s test *p* = 0.022, Egger’s test *p* = 0.004). However, adjusting the model by the trim and fill method did not influence the conclusions (OR = 1.23, 95% CI = 1.02–1.48) (Figure 3).

### 2.5. Discussion

The meta-analysis revealed that *eNOS 4b/a* polymorphisms were significantly associated with CAD in three genetic models (dominant model, homozygote comparison, and heterozygote comparison). These data suggest that carriers of the *a* allele of the *eNOS 4b/a* gene might be predisposed to CAD. Heterogeneity was observed in these three models; however, subsequent meta-regression analyses revealed that case and total sample size could explain the heterogeneity. Subgroup analysis based on source of the control population revealed that population-based subgroups with the *aa* or *ab* genotypes had a significantly increased risk of CAD. In stratified analyses according to ethnicity, significantly increased risk was detected in the African subgroup with the *aa* or *ab* genotypes and the Asian subgroup with the *aa* genotype. Positive associations were also identified in studies whose case or control sample size was <500 with the *aa* or *ab* genotypes. Similarly, significant associations were identified in studies with the *aa* or *ab* genotypes in a subgroup analysis according to MI.

In 1998, a Japanese study including 413 subjects reported that the plasma levels of mono-nitrogen oxides in the *a* allele group (31.2 ± 2.00 μmol/L) were significantly lower than in the *b* allele group (35.5 ± 0.93 μmol/L) [48]. Reduced plasma concentrations of nitrogen oxides (NO) in carriers of the *a* allele were also identified in a study by Rittig *et al.* in a German population [49]. Prevailing experimental and clinical data suggest that decreased NO bioavailability accelerates the progression of atherosclerosis presumably through mechanisms such as platelet activation, vascular smooth muscle proliferation, leukocyte adhesion to the endothelium and increased vascular production of reactive oxygen species [50,51]. Therefore, it is possible that *eNOS 4b/a* gene polymorphisms could contribute to CAD risk by decreasing NO production, which subsequently affects the development of CAD. However, because this variant is located in the intronic region, it is possible that it is in linkage disequilibrium with other functional variants in regulatory regions of the *NOS* gene. In 2002, Wang *et al.* demonstrated that the *eNOS 4a* allele coordinated with the T-786C variant in the promoter region to regulate the transcriptional efficiency of *eNOS* in a haplotype-specific fashion [52]. In 2013, Narne *et al.* reported that the *eNOS 4b/a* polymorphism affected the bioavailability and activity of NO via linkage disequilibrium with T-786C and G894T variants [25]. Consistent with these observations, our meta-analysis revealed that individuals carrying the *eNOS 4a* allele had a higher CAD risk than subjects carrying the *4b* allele.

Our results also suggested that the *4a* allele might be a risk factor for CAD among population-based but not hospital-based subgroups. One explanation for this is that there were differences in the frequency of exposure to risk factors between hospital- and population-based studies. The use of hospital-based population might limit the general applicability of the data. Furthermore, the sample size and number of studies in the hospital-based group might not be sufficient to evaluate potential associations.

In the subgroup analysis according to ethnicity, an increased risk in *4a* carriers was identified among African and Asian populations but not Caucasians. The results could be explained by varying susceptibility of different ethnicities to the *eNOS 4b/a* polymorphism among different ethnicities. Environmental characteristics might also contribute to this discrepancy.

Two previous meta-analyses assessing *eNOS 4b/a* and CAD risk have been performed. In 2004, one study reported an association between *eNOS 4a/a* and ischemic heart disease compared with *eNOS 4b/b* [10]. Although IHD and CAD have overlapping pathogeneses, they are different disease. A second meta-analysis performed in 2010 did not reveal any association between this *eNOS* genetic variant and CAD risk [11]; therefore, our results contradict this study. This could be explained by several reasons. First, different inclusion and exclusion criteria were used. We excluded four studies in which the control population deviated from Hardy-Weinberg equilibrium (HWE) [53–56]. We also incorporated novel data published between 2010 and October 2013 in our study. Therefore, the number and content of the studies included by Li *et al.* and in the current meta-analysis differed. Second, we performed overall analyses in four genetic models; in contrast Li *et al.* did not perform homozygote comparison and heterozygote comparisons. We also performed subgroup analysis based on ethnicity, the source of the control population, and case sample size to identify all possible associations between *4b/a* polymorphisms and CAD; these factors were not investigated previously.

The present study has some limitations. First, heterogeneity was detected in three genetic models, suggesting that results should be interpreted with caution. Second, most of the included studies were case-control studies, where the cases were survivors of cardiovascular events; those who did not survive were not enrolled. Finally, the sample size in the studies was inadequate. The insufficient sample size might increase the probability of false positives or false negatives.

## 3. Experimental Section

### 3.1. Literature Search

We systematically searched PubMed using the following medical subject headings (MeSH) or search terms: (“CHD” OR “atherosclerosis” OR “coronary atherosclerosis” OR “aortic atherosclerosis” OR “coronary heart disease” OR “coronary artery disease” OR “coronary disease” OR “myocardial infarction” OR “MI” OR “acute coronary syndrome”) and (“polymorphism*” OR “variation”) and (“eNOS” OR “endothelial nitric oxide synthase”). The search covered all English language publications within a range of published years from 1996 to 2013 (the last search was performed on 31 October 2013). The bibliographies of previous meta-analyses and published reviews were also checked for additional relevant publications.

### 3.2. Inclusion and Exclusion Criteria

The inclusion criteria for studies were as follows: (1) independent case-control, cohort or cross-sectional studies; (2) studies that evaluated the association between *eNOS* intron *4b/a* variable number of tandem repeats (VNTR) polymorphisms and CAD susceptibility; (3) studies that provided complete data regarding genotype number and allele frequencies; (4) studies in which CAD was diagnosed based on coronary angiography as well as clinical criteria with clearly reported details; (5) studies that also included controls demonstrated to lack CAD either by coronary arteriography or by clinical criteria; and (6) studies that were published in English. The exclusion criteria were as follows: (1) insufficient data regarding genotypes and allele frequencies; (2) unconfirmed diagnosis of CAD; (3) the genotype distribution of the control population did not conform to HWE; and (4) overlapping publications.

### 3.3. Data Extraction

Two researchers (Y.Y. and K.D.) extracted information including the first author, year of publication, country, ethnicity of the study population, source of controls, genotype number in cases and controls, HWE in controls and study outcome. Data were extracted separately and compared to reach a consensus. Different ethnicities were categorized as Asians and non-Asians (Caucasians, Africans, or others). Different ethnic descents in one study were considered to be independent studies when subanalyses based on ethnicity were performed.

### 3.4. Statistical Analysis

The association between *eNOS 4b/a* gene polymorphisms and CAD was compared using the odds ratio (OR) corresponding to a 95% confidence interval (95% CI). We used four different genetic models in our analysis: homozygote comparison (*aa vs. bb*), heterozygote comparison (*ab vs. bb*), dominant (*aa* + *ab vs. bb*), and recessive models (*aa vs. ab* + *bb*).

Heterogeneity between studies was assessed using *p* values for the Q statistic and *I*^2^ values. Significance was set at the *p* < 0.1 level; *p* < 0.1 for the Q test indicated there was heterogeneity across the studies and *p* > 0.1 for the *Q* test indicated a lack of heterogeneity among studies. The random-effects model was used to estimate the pooled OR according to the DerSimonian and Laird method [57]. *I*^2^ was calculated to describe the percentage of variation caused by the heterogeneity: 0%–25%, no heterogeneity; 25%–50%, moderate heterogeneity; 50%–75%, large heterogeneity; and 75%–100%, extreme heterogeneity).

To determine the source of heterogeneity across studies, logistic meta-regression analyses were performed. Publication year, population ethnicity, source of the control group, outcome, total sample size, case sample size, control sample size, and the ratio of case size to control size were examined to evaluate the extent to which different variables explained any observed heterogeneity. Stratified analyses were also performed for ethnicity, source of controls, and case sample size. Sensitivity analyses were conducted by removing each study individually and consecutively from the combined data set. Re-analysis of the remaining studies, allowed the robustness of the main findings to be tested. In addition, a funnel plot was used to estimate the potential publication bias; Begg’s and Egger’s tests were then used to examine funnel plot asymmetry with significance set at *p* < 0.05, and the trim and fill method was performed when bias was detected. STATA 12.0 software was used to perform all statistical analyses (StataCorp, College Station, TX, USA).

## 4. Conclusions

In conclusion, the results of the current meta-analysis suggested that *eNOS 4b/a* polymorphisms could increase the risk of CAD, particularly in African populations and among the population-based studies. Further studies with larger sample sizes should be performed to confirm these findings.

## Supplementary Information



## Figures and Tables

**Table 1.** Study Characteristics of genotypes in coronary artery disease (CAD) cases and controls in the analysis of endothelial nitric oxide synthase (*eNOS*) *4b/a* polymorphism.

Author	Year	Country	Ethnicity	Source of controls	End points	Sample size		Genotype distribution					

								Control			Case		

						Control	Case	*aa*	*ab*	*bb*	*aa*	*ab*	*bb*
Wang *et al.*	1996	Australia	Caucasian	PB	CAD	153	549	1	50	102	18	122	409
Hibi *et al.*	1998	Japan	Asian	PB	AMI	357	226	5	68	284	4	48	174
Ichihara *et al.*	1998	Japan	Asian	HB	MI	550	455	7	97	446	9	107	339
Odawara *et al.*	1998	Japan	Asian	HB	CHD	122	42	0	19	103	0	11	31
Hooper *et al.*	1999	American	African	PB	MI	185	110	15	68	102	14	52	44
Nakagami *et al.*	1999	Japan	Asian	HB	CAD	34	40	1	9	24	1	18	21
Park *et al.*	2000	Korea	Asian	PB	AMI	206	121	1	49	156	5	23	93
Fowkes *et al.*	2000	UK	Caucasian	PB	CHD	300	137	5	64	231	4	37	96
Pulkkinen *et al.*	2000	Finland	Caucasian	PB	CHD	110	308	5	26	79	9	96	203
Sigusch *et al.*	2000	Germany	Caucasian	HB	CHD	413	625	11	115	287	12	159	454
Granath *et al.*	2001	Australia	Caucasian	PB	CAD	620	567	14	158	448	11	138	418
Alvarez *et al.*	2001	Spain	Caucasian	PB	CAD	300	170	6	72	222	2	37	131
Hwang *et al.*	2002	Taiwan	Asian	HB	CAD	70	149	0	14	56	1	32	116
Gardemann *et al.*	2002	Germany	Caucasian	PB	MI	528	1265	13	144	371	25	306	934
Cine *et al.*	2002	Turkey	other	PB	MI	306	207	2	55	249	9	55	143
Park *et al.*	2004	Korea	Asian	HB	ACS	142	164	7	30	105	4	21	139
Agema *et al.*	2004	Netherlands	Caucasian	PB	CAD	466	752	8	77	381	12	195	545
Letonja *et al.*	2004	Slovenia	Caucasian	PB	CAD	109	151	6	30	73	6	41	104
Fatini *et al.*	2004	Italy	Caucasian	PB	ACS	537	477	14	138	385	24	138	315
Milutinovic *et al.*	2005	Slovenia	Caucasian	NM	CAD	188	215	10	58	120	11	60	144
Rao *et al.*	2005	America	Caucasia + African	HB	CAD	50	144	1	17	32	6	40	98
Matyar *et al.*	2005	Turkey	other	HB	CAD	133	133	1	35	97	5	47	81
Salimi *et al.*	2006	Iran	other	NM	CAD	158	141	1	29	128	3	41	97
Agirbasli *et al.*	2006	Turkey	other	HB	CAD	100	100	0	21	79	4	21	75
Kim *et al.*	2007	Korea	Asian	PB	CAD	211	147	0	40	171	2	35	110
Rios *et al.*	2007	Brazil	Caucasia + African	HB	CAD	267	323	12	90	165	18	101	204
Sampaio *et al.*	2007	Brazil	other	HB	AMI	103	115	4	32	67	4	29	82
Vasilakou *et al.*	2008	Greece	Caucasian	PB	CAD	161	209	5	39	117	9	60	140
Meluzin *et al.*	2009	Czech rep	Caucasian	HB	CAD	89	321	0	26	63	10	103	208
Kincl *et al.*	2009	Czech rep	Caucasian	HB	CAD	222	939	9	63	150	35	272	632
Bae *et al.*	2010	Korea	Asian	PB	CAD	196	192	0	35	161	2	42	148
Gururajan *et al.*	2010	India	Asian	NM	ACS	100	106	4	19	77	12	36	58
Jaramillo *et al.*	2010	Chile	other	PB	CAD	112	112	1	16	95	3	12	97
Jemaaa *et al.*	2012	Tunisia	African	PB	MI	250	310	4	61	185	15	105	190
Da Costa Escobar Piccoli *et al.*	2012	Brazil	other	PB	ACS	108	132	6	34	68	14	31	87
Kallel *et al.*	2013	Tunisia	African	PB	MI	225	303	3	58	164	15	101	187
Narne *et al.*	2013	India	Asian	HB	CAD	121	160	2	42	77	5	50	105

Abbreviations: eNOS, endothelial nitric oxide synthase; HB, hospital-based; PB, population-based; NM, not mentioned; OR, odds ratios; CI, confidence intervals; CAD, coronary artery disease; CHD, coronary heart disease; MI, myocardial infarction; AMI, acute myocardial infarction; and ACS, acute coronary syndrome.

**Table 2.** Pooled odds ratios (OR) and 95% confidence intervals (CI) of the association between *eNOS 4b/a* polymorphism and CAD.

Total and subgroups	Studies	Homozygote comparison (*aa vs. bb*)			Heterozygote comparison (*ab vs. bb*)			Dominant model (*aa* + *ab vs. bb*)			Recessive model (*ab* + *bb vs. aa*)		

		OR (95% CI)	*p*	*I*^2^ (%)	OR (95% CI)	*p*	*I*^2^ (%)	OR (95% CI)	*p*	*I*^2^ (%)	OR (95% CI)	*p*	*I*^2^ (%)
Total	37	1.47 (1.16–1.87)	0.085	25.5	1.14 (1.02–1.27)	<0.001	56.7	1.18 (1.06–1.33)	<0.001	61.1	0.72 (0.58–0.89)	0.213	15.4
MI	6	2.30 (1.17–4.51)	0.020	62.6	1.42 (1.07–1.89)	0.002	74.2	1.50 (1.09–2.07)	<0.001	80.7	0.56 (0.39–0.82)	0.054	54.1

**Ethnicity**													

Caucasian	16	1.03 (0.80–1.31)	0.621	0.0	1.04 (0.90–1.20)	0.006	53.3	1.04 (0.91–1.19)	0.007	53.0	0.98 (0.77–1.25)	0.679	0.0
Asian	11	1.82 (1.03–3.20)	0.316	13.8	1.21 (0.96–1.54)	0.027	50.5	1.26 (0.99–1.61)	0.011	56.3	0.60 (0.36–0.99)	0.434	0.4
African	5	2.93 (1.71–5.03)	0.821	0.0	1.46 (1.16–1.83)	0.353	9.4	1.58 (1.27–1.96)	0.370	6.5	0.41 (0.24–0.70)	0.684	0.0
Other	7	2.65 (1.37–5.13)	0.369	7.8	1.17 (0.84–1.62)	0.033	56.4	1.29 (0.94–1.77)	0.033	56.3	0.39 (0.21–0.71)	0.511	0.0

**Source of control**													

HB	14	1.11 (0.78–1.56)	0.509	0.0	1.04 (0.88–1.23)	0.072	38.2	1.07 (0.89–1.27)	0.027	46.8	0.90 (0.64–1.26)	0.613	0.0
PB	20	1.64 (1.18–2.27)	0.070	33.9	1.16 (1.00–1.35)	<0.001	61.8	1.21 (1.05–1.40)	<0.001	63.4	0.65 (0.48–0.89)	0.113	28.7
NM	3	2.05 (0.66–6.32)	0.111	54.4	1.54 (0.80–2.98)	0.011	77.6	1.63 (0.80–3.30)	0.003	82.6	0.58 (0.24–1.40)	0.232	31.6

**Case size**													

>500	6	0.88 (0.62–1.24)	0.666	0.0	0.96 (0.74–1.25)	<0.001	78.7	0.97 (0.76–1.22)	0.001	75.9	1.14 (0.80–1.60)	0.642	0.0
<500	31	1.75 (1.35–2.28)	0.262	13.2	1.20 (1.07–1.35)	0.011	40.7	1.26 (1.11–1.42)	0.002	48.0	0.61 (0.48–0.77)	0.453	0.8

**Control size**													

>500	4	1.21 (0.72–2.05)	0.138	45.6	1.07 (0.85–1.36)	0.024	68.3	1.09 (0.84–1.42)	0.007	75.1	0.84 (0.52–1.34)	0.214	33.1
<500	33	1.56 (1.19–2.05)	0.113	23.9	1.15 (1.01–1.31)	<0.001	55.6	1.20 (1.06–1.36)	<0.001	59.0	0.68 (0.53–0.88)	0.233	14.8

Abbreviations: eNOS, endothelial nitric oxide synthase; OR, odds ratios; CI, confidence intervals; MI, myocardial infarction; HB, hospital-based; PB, population-based; and NM, not mentioned.
