# Supplementary material for: Endothelial Nitric Oxide Synthase (eNOS) 4b/a Gene Polymorphisms and Coronary Artery Disease: Evidence from a Meta-Analysis
Source: Int J Mol Sci. 2014 May 7;15(5):7987–8003. doi: 10.3390/ijms15057987 (PMC4057714; doi:10.3390/ijms15057987)
Supplement: Supplementary file 1 [file ijms-15-07987-s001.pdf]

Supplementary Information

Figure S1. Forest plot of CAD risk associated with the eNOS 4ba polymorphism by ethnicity (homozygote comparison).

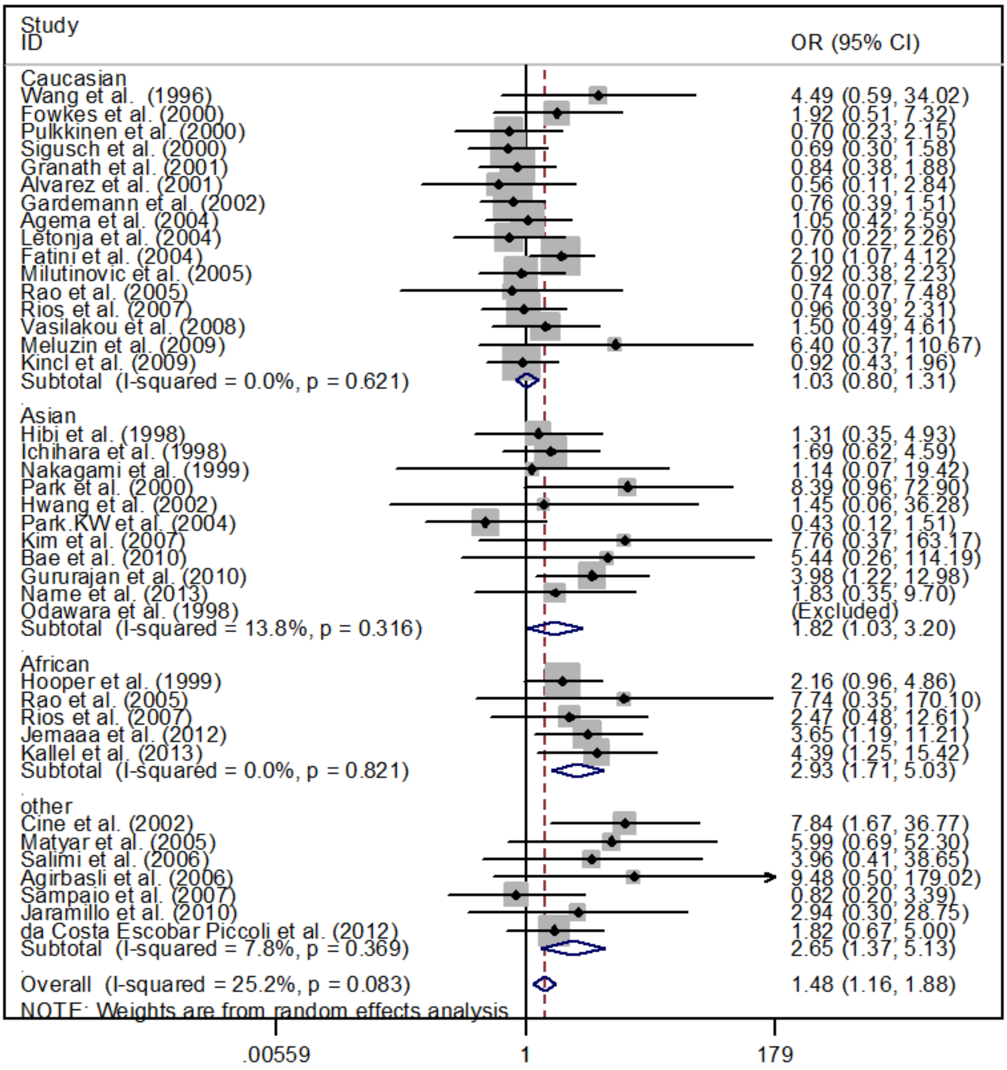

Figure S2. Influence analysis of the summary odds ratio coefficients on the association for the eNOS 4ba genotype with CAD risk.

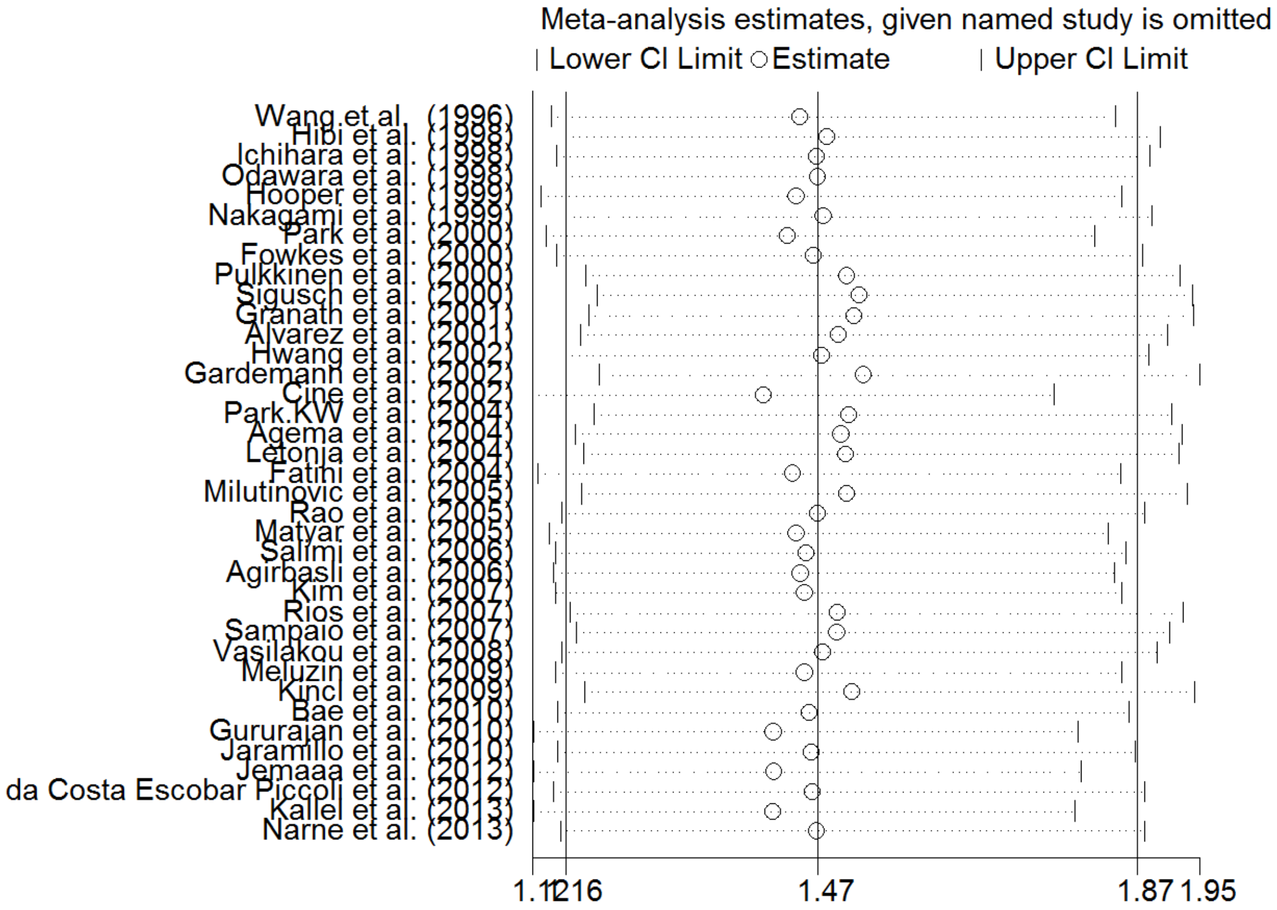

© 2014 by the authors; licensee MDPI, Basel, Switzerland. This article is an open access article distributed under the terms and conditions of the Creative Commons Attribution license (http://creativecommons.org/licenses/by/3.0/).
